# Pott disease: Vertebral Tuberculosis

**DOI:** 10.1590/0037-8682-0491-2020

**Published:** 2021-03-08

**Authors:** Libardo Valencia Chicué, Indalecio Carboni Bisso, Marcos Las Heras

**Affiliations:** 1 Hospital Italiano de Buenos Aires, Terapia Intensiva de Adultos, Buenos Aires, Argentina.; 2 Sanatorio Franchin, Terapia Intensiva de Adultos, Buenos Aires, Argentina.

A 25-year-old man with no medical record but a history of recent imprisonment before consultation was hospitalized for acute lower limb paresthesias and severe back pain. His relatives reported that he had presented with asthenia, weight loss, and dry cough with intermittent episodes of hemoptysis in the last five months.

**FIGURE 1:**
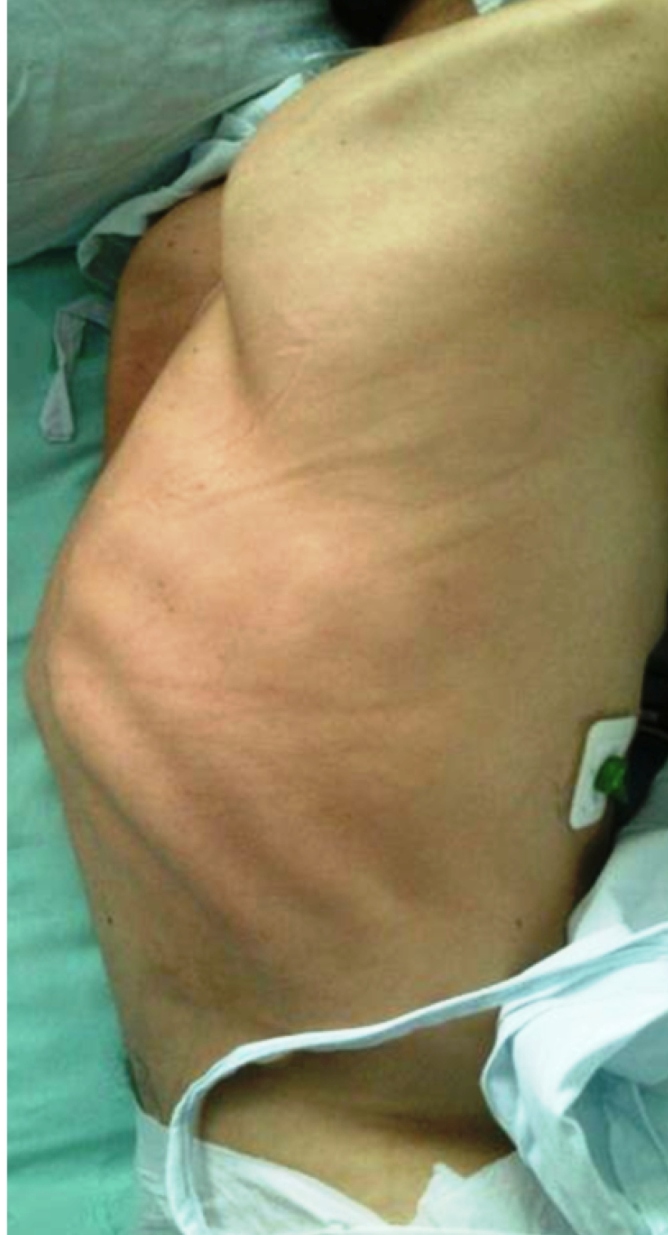
Thoracic kyphosis.

**FIGURE 2:**
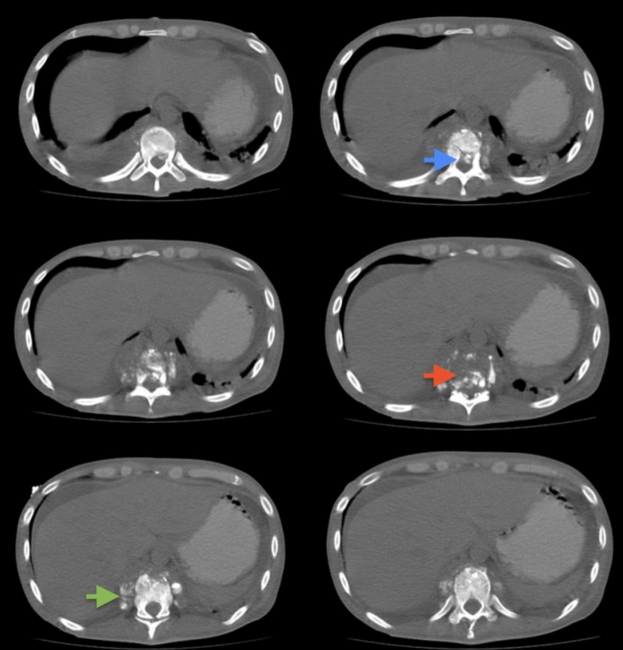
Chest CT scan: T10 and T11 vertebral destruction (red arrow) with a thickening of the paravertebral soft tissues (green arrow) and the displacement of bone fragments into the medullary canal (blue arrow).

**FIGURE 3:**
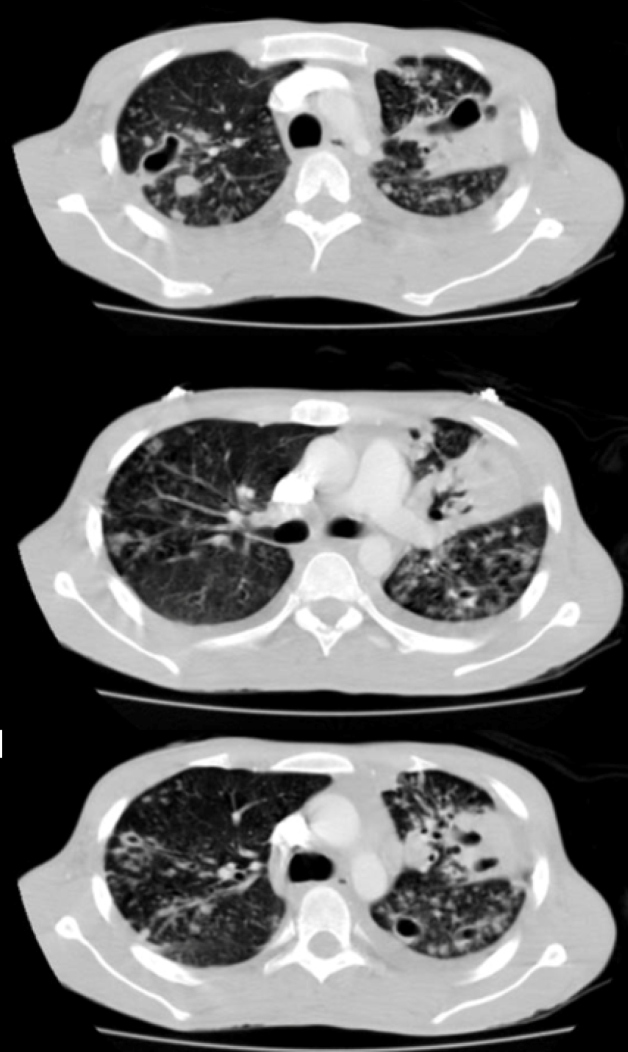
Chest computed tomographic scan showing thick-walled cavitated lesions in the upper lobes.

A physical examination revealed evidence of thoracic kyphosis and pain on superficial palpation (Figure 1). A chest computed tomography reported vertebral destruction at T10 and T11 with displacements of bone fragments towards the medullary canal (Figure 2). In addition, multiple pulmonary caverns were detected (Figure 3). Later, a diagnosis of disseminated tuberculosis with pulmonary and vertebral compromise (Pott disease) was made, and concomitant immunosuppression was ruled out. Vertebroplasty and fixation were planned as the bone cultures were positive for *Mycobacterium tuberculosis*. He received treatment with isoniazid, pyrazinamide, rifampicin, and ethambutol for six months after which he had an adequate clinical response.

In general, skeletal muscle involvement occurs in 10 to 35% of cases of extrapulmonary tuberculosis^1^, with the lower thoracic and upper lumbar vertebrae being more frequently affected. Kyphosis carries a high risk of spinal compression as it results in neurological disorders such as paresthesias, paresis, and even paraplegia^2^. Diagnostic confirmation is made by biopsy along with clinical and radiological findings. Usually, the use of tuberculostatic drugs along with adequate nutritional support is the cornerstone of treatment, showing clinical improvement in up to 90% of cases treated in a multimodal manner^3^.
